# Coalescent Simulations Reveal Hybridization and Incomplete Lineage Sorting in Mediterranean *Linaria*

**DOI:** 10.1371/journal.pone.0039089

**Published:** 2012-06-29

**Authors:** José Luis Blanco-Pastor, Pablo Vargas, Bernard E. Pfeil

**Affiliations:** 1 Departamento de Biodiversidad y Conservación, Real Jardín Botánico (RJB-CSIC), Madrid, Spain; 2 Department of Biological and Environmental Sciences, Gothenburg University, Gothenburg, Sweden; Aarhus University, Denmark

## Abstract

We examined the phylogenetic history of *Linaria* with special emphasis on the Mediterranean sect. *Supinae* (44 species). We revealed extensive highly supported incongruence among two nuclear (ITS, AGT1) and two plastid regions (*rpl32-trnL^UAG^*, *trnS-trnG*). Coalescent simulations, a hybrid detection test and species tree inference in *BEAST revealed that incomplete lineage sorting and hybridization may both be responsible for the incongruent pattern observed. Additionally, we present a multilabelled *BEAST species tree as an alternative approach that allows the possibility of observing multiple placements in the species tree for the same taxa. That permitted the incorporation of processes such as hybridization within the tree while not violating the assumptions of the *BEAST model. This methodology is presented as a functional tool to disclose the evolutionary history of species complexes that have experienced both hybridization and incomplete lineage sorting. The drastic climatic events that have occurred in the Mediterranean since the late Miocene, including the Quaternary-type climatic oscillations, may have made both processes highly recurrent in the Mediterranean flora.

## Introduction

Gene trees can differ from one another and do not always correspond to species trees [1]–[4]. Wendel and Doyle [5] listed three categories of processes that may cause incongruent patterns: technical causes, organism-level processes and gene- or genome-level processes. If technical causes, selection, paralogy and recombination can be ruled out, then (i) hybridization among fully differentiated species with subsequent fixation of nuclear and/or organellar loci and (ii) the incomplete random sorting of alleles at many loci independently due to short intervals between divergence events (hereafter incomplete lineage sorting) often remain as the main hypotheses that can explain gene tree incongruence [6]–[11]. Typically, phylogenetic analyses using single locus datasets (e.g. [12]–[14]) or concatenated datasets (e.g. [15]–[18]) have provided inferences of relationships in numerous plant groups. Nonetheless, a tree based on a single locus or concatenated genes may lead to a spurious representation of the history of the species [19], [20]. Several methods that distinguish hybridization from incomplete lineage sorting have been recently described [21]–[23]. However, many independent loci are needed for their implementation and hybridization is difficult to uncover if multiple reticulation events have occurred. Ané *et al.* [24] implemented a method that can accommodate any source of incongruence even using a limited number of loci, but this method is unable to determine the process causing incongruence among phylogenies. Also, Maureira-Butler *et al.* [6] and Joly *et al.* [10] have proposed statistical frameworks, applicable to datasets with few independent loci, where hybridization can be detected in the presence of incomplete lineage sorting. Alternatively, several models can estimate the correct species tree if incongruence is due to incomplete lineage sorting alone [20], [25]–[29], but in such models hybridization signals need to be previously ruled out or excluded. If not, an incorrect species tree may be inferred by such methods [20], [30].

Both polyploid and homoploid hybrid speciation might represent a large fraction of the source of plant biodiversity on Earth [31]. In the Mediterranean basin, several plant groups suffered secondary contacts in their postglacial colonization routes from their glacial maximum refugia located in southern peninsulas [32] or after altitudinal migrations in restricted areas within peninsulas (e.g. Iberian Peninsula, [33], [34]). A considerable proportion of the present Mediterranean plant diversity may be the result of hybridization episodes, which *per se* represent a challenge for phylogenetic reconstruction. Besides this, species complexes that underwent rapid speciation also represent a major challenge for molecular systematics. In those groups species relationships could be obscured by the ancestral polymorphisms retained through speciation events as a consequence of incomplete lineage sorting [2], [35]. In the Mediterranean region, rapid plant speciation has been recently detected [36]–[38] and associated with adaptation to the establishment of the Mediterranean climatic rhythm (summer drought) (3.2 Ma) or the Quaternary-type Mediterranean climatic fluctuations (2.3 Ma) [39].

Toadflaxes (*Linaria* Mill.) constitute the largest genus within the snapdragon lineage (tribe Antirrhineae). *Linaria* comprises c.150 species that are widely distributed in the Palearctic region, but the genus is most diverse in the Mediterranean basin. The origin of the genus has been placed in the Miocene [40] predating the Messinian Salinity Crisis [41]. The monophyly of *Linaria* has been suggested based on nrDNA (ITS) sequences of eight species representing all sections [42], however, whether the sections constitute natural groups remains uncertain. Numerous taxonomic treatments of *Linaria* have been proposed [43]–[51], but remarkable disagreement in the infrageneric classification suggests complex evolutionary processes. The latest classification of the genus recognizes seven sections (*Linaria, Speciosae, Diffusae, Supinae, Pelisserianae, Versicolores* and *Macrocentrum*) [45]. Section *Supinae* (Benth.) Wetts. (hereafter *Supinae*) is a clear example of the systematic complexity within *Linaria* because of the disagreement in taxonomic treatments (Table 1). *Supinae* comprises 44 diploid (2n = 12) [52] hermaphroditic annual and perennial species differentiated from other sections by their laterally-compressed winged seeds that have a horizontal arrangement in globose capsules [45]. *Supinae* species are distributed in the temperate regions of Europe, northern Africa and western Asia (circum-Mediterranean distribution), with the highest diversity found in the Iberian Peninsula (40 species) [44], [45].

**Table 1:** Systematic classification of *Linaria* sect *Supinae* suggested in this study and its relation with previous classifications.

Bentham (1846)	Wettstein (1895)	Valdés (1970) and Viano (1978)	Sutton (1988), Sáez (2008)	Present study
*Linaria* sect. *Linariastrum* Chav.	*Linaria* Juss.	*Linaria* Miller	Sect. *Supinae* (Bentham) Wettst.	Sect. *Supinae* (Bentham) Wettst.
§ *Arvenses* Bentham p.p.max.	Sect. *Arvenses* (Bentham) Wettst. p.p.max.	Sect. *Arvenses* (Bentham) Wettst.	Subsect. *Supinae* p.p.	Subsect. *Arvenses*
§ *Supinae* Bentham p.p.§ *Diffusae* Bentham p.p.§ *Grandes* Benthamp.p.min.	Sect. *Supinae* (Bentham)Wettst. p.p.	Sect. *Supinae* (Bentham) Wettst.subsect. *Supinae*	Subsect. *Supinae* p.p.	Subsect. *Supinae*
§ *Supinae* Bentham p.p.§ *Versicolores* Bentham p.p.min.	Sect. *Supinae* (Bentham)Wettst. p.p.	Sect. *Supinae* subsect. *Saxatile* Valdésp.p. max Sect. *Supinae* (Bentham) Wettst.subsect. *Supinae* p.p. Sect. *Supinae* subsect.*Amethystea* Valdés Sect. *Bipunctatae* Vianop.p.max.	Subsect. *Saxatile* Valdés Subsect. *Supinae* p.p.	Subsect. *Saxatile* Valdés
–	–	–	Subsect. *Trimerocalyx* (Murb.) D.A. Sutton	–

p.p. = *pro parte*.

p.p.max = *pro parte maxima*.

p.p.min = *pro parte minima*.

In *Linaria*, hybrid species have been historically described when intermediate characters of two species meet in a plant [53], [54]. In section *Supinae* several natural hybrids have been previously reported [44], [55]–[57]. Artificial experiments have also shown the potential of hybridization inasmuch as *Supinae* species that do not meet in nature can produce capsules after hand cross-pollination ((Blanco-Pastor, unpublished), [53]). The highest fertilization success was found in crosses among *Supinae* species (13 successful crosses of 20 assayed), followed by clearly lower values in inter-sectional crosses (four successful crosses of 14) [53]. A lack of internal reproductive barriers among *Supinae* species is then suggested. Despite this, external barriers such as allopatry do exist at the present time within *Supinae* as few species have overlapping distributions. However, such geographical barriers may have not existed during glaciations.

The high chance for hybridization in *Linaria* may affect phylogenetic reconstruction in this genus. Nonetheless, incomplete lineage sorting cannot be discarded as a cause of phylogenetic incongruence. Both processes can be difficult to distinguish, but may also occur simultaneously [58]. Within this framework, we investigate causes of incongruence between three presumably unlinked loci. Two nuclear (ITS and AGT1) and two linked plastid (*rpl32-trnL^UAG^* and *trnS-trnG*) regions are herein sequenced for *Linaria*, with special emphasis in *Supinae* species. Our aims are: (i) to test for the presence of reticulation signals by simulations under the coalescent model using the method of Maureira-Butler *et al.* [6], (ii) to detect individuals that may have been affected by historical hybridization (hereafter potential hybrids), (iii) to exclude potential hybrids and infer the species tree using a method that accounts for incomplete lineage sorting (*BEAST) [20], (iv) to compare the *BEAST species tree with our original gene trees to identify random sorting episodes, and (v) to recover the reticulation events by locating the parental lineages of the potential hybrids in a multilabelled species tree. The ultimate goal is to disclose the evolutionary history of *Supinae* by exploring the presence of incomplete lineage sorting and/or reticulation events that may have occurred during the course of the evolution of this plant group.

## Materials and Methods

### Sampling Strategy

Individuals were collected in the field and dried in silica gel or obtained from herbaria (MA, E, RNG) (Table S1). Total genomic DNA was extracted using the Dneasy Plant Mini Kit (QUIAGEN Inc., California). We amplified (using an Eppendorf Mastercycler Epgradient S, Westbury, NY) a low copy nuclear gene intron (AGT1) [59], the nuclear ribosomal internal transcribed spacer (ITS) [60] and two plastid regions (*rpl32-trnL^UAG^*, *trnS-trnG*) [61], [62] in 52 individuals representing 46 *Linaria* species plus one individual of *Antirrhinum* and one individual of *Chaenorhinum*. In particular, we used one species of sect. *Macrocentrum* (*L. chalepensis*), three species of sect. *Versicolores* (*L. spartea, L. gharbensis, L. multicaulis*), five species of sect. *Linaria* (*L. meyeri, L. loeselii, L. odora, L. thibetica, L. vulgaris*), four species of sect. *Speciosae* (*L. ventricosa, L. dalmatica, L. peloponnesiaca, L.* *genistifolia*), seven species of sect. *Diffusae* (*L. albifrons, L. flava, L.* *triphylla, L. laxiflora, L. warionis, L. haelava, L. joppensis*) and 24 of the 44 species of section *Supinae* [45]. We followed Sutton’s species delimitation [45] for the non-Iberian species and Sáez & Bernal’s delimitation [44] for the Iberian species but with minor changes regarding the “*Linaria verticillata* group” and the “*Linaria alpina* group” [44], [63] (see Methods S1). We also included one additional species neither considered by Sutton nor Sáez & Bernal: *L. almijarensis* Campo & Amo [64] (see Table S1). All necessary permits were obtained for the described field studies. In cases where plant locations were protected we obtained permissions from the "Consejería de Medio Ambiente" of Andalusian Government (Spain), references: GB-86/2010/EA/FL/FA/JMLV, ENSN/JSG/IHC/MCF. Amplification products were outsourced for sequencing to a contract sequencing facility (Macrogen, Seoul, South Korea) on an ABI Prism® 3730xi DNA sequencer, using the same primer set as for PCR. Sequence data were edited using Geneious software (Biomatters Ltd., Auckland, New Zealand). Sequences are available in GenBank (see Table S1).

**Figure 1:**
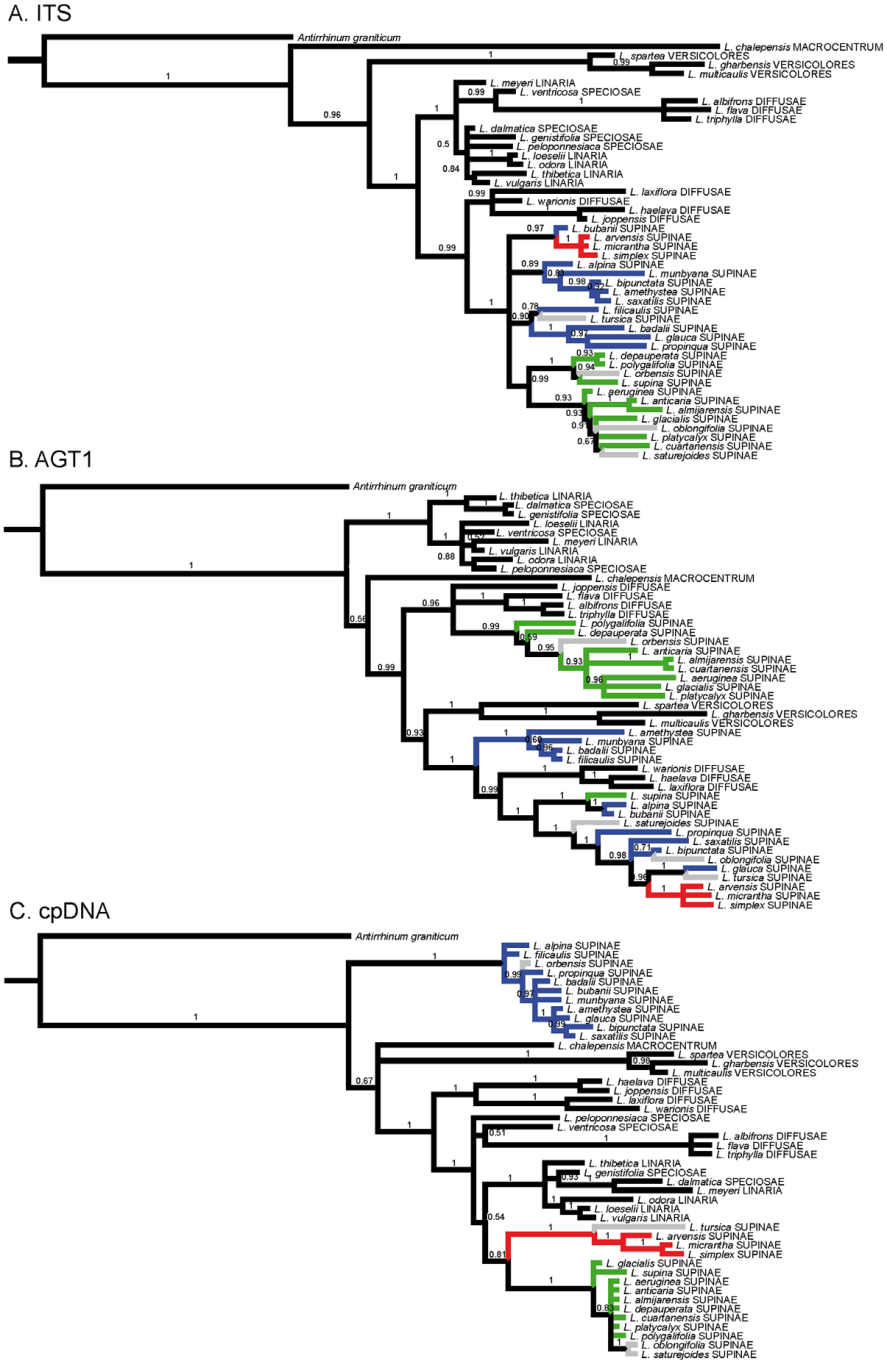
Gene trees. Phylogenetic relationships of 47 samples representing 46 *Linaria* species and one individual of *Antirrhinum* as the outgroup. One species of sect. *Macrocentrum*, three species of sect. *Versicolores*, five species of sect *Linaria*, four species of sect. *Speciosae* and 28 species of sect. *Supinae* are represented. 50% Mayority-rule consensus tree obtained in the Bayesian analysis of ITS (A), AGT1 (B) and cpDNA (C) sequences are shown. Numbers above branches represent Bayesian posterior probabilities. Phylogenetic trees are based on one sample and one allele per species, when the two alleles were not sister we used the most incongruent one respecting the other two genes. *Linaria* sections following Sutton [45] are shown in capital letters. Colors represent the systematic nomenclature for *Supinae* clades as suggested in this paper (see Fig. 4). Species with key traits from two *Supinae* clades (Fig. 4) are represented in grey.

### Deciphering of Haplotypes in Unphased Genotypes

More than one allele was found in both AGT1 and ITS in Sanger sequenced PCR amplicons. To decipher these, we first estimated the gametic phases of the sequences using Arlequin 3.5.1.2 [65]. This program performs a Gibbs sampling via the ELB algorithm [66] to obtain the posterior probability of phased haplotypes. The settings for the ELB algorithm were as follows: dirichlet alpha value: 0.01, epsilon value: 0.1, heterozygote site influence zone: 5, gamma value: 0.01, sampling interval: 500, no. of samples: 2000, burn-in steps: 100000 and 0% of recombination steps. AGT1 haplotypes retrieved with posterior probability under 0.95 were confirmed by cloning the purified PCR products using the Promega Corporation protocol (Madison, USA) with JM109 High Efficiency competent cells and pLysS plasmids. Four single recombinant colonies from each reaction were screened. Amplifications were performed using the T7-SP6 plasmid primers. All ITS haplotypes inferred with Arlequin were used to build allele trees. In only one case (*L. bubanii)* ITS haplotypes were not inferred as sister (or very closely related) sequences in the gene trees. As the phase posterior probability for this individual was low (0.41), we empirically confirmed the *L. bubanii* ITS haplotypes by sequencing the PCR product using allele-specific primers as described in Scheen *et al.* [67].

### Test for Recombination

Recombination was tested within ITS and AGT1 datasets using RDP 3.44 [68] with the following methods: RDP [69], Geneconv [70], MaxChi [71], Bootscan/Recscan [72], SisScan [73], 3Seq [74] and Chimaera [75]. We selected 0.05 as the p-value cut-off in general settings and internal references only in the RDP method. A window size of 150 and step size of 20 was used in the Bootscan and SisScan methods and a variable window size was set in MaxChi and Chimaera methods. We considered that recombination was likely if it was accepted by more than two methods. For the remaining settings we used the default values.

**Figure 2:**
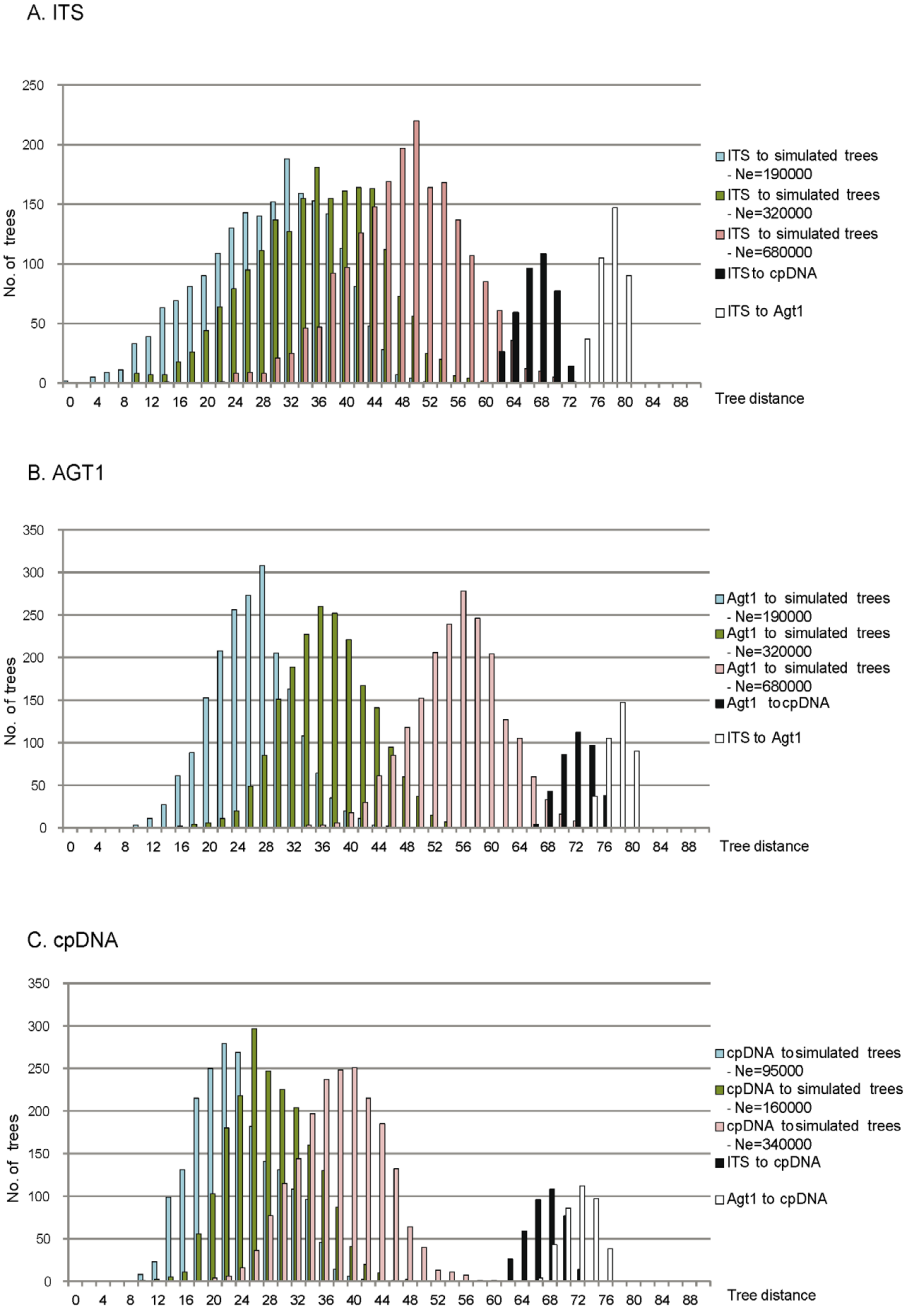
Baseline and observed distributions of tree distances. Frequency distribution of tree-to-tree distances between 20 representative trees from the stable posterior distribution of the Bayesian analysis (ITS (A), AGT1 (B) and cpDNA (C)) and 100 simulated gene trees obtained by coalescent simulations (baseline distributions). Blue, green and red bars represent baseline distributions under *L. glacialis*, *L. elegans* and *L. simplex* N_e_ estimates respectively. Black and white bars represent the distances between gene trees (observed distributions).

### Gene Trees Estimation and Calculation of Dates

The haplotype sequences obtained from the three datasets (ITS, AGT1, cpDNA) were analyzed by Bayesian Inference in MrBayes 3.1.2 [76] after alignment with MAFFT v.6 [77] (with corrections by visual inspection) and optimal substitution model selection in jModeltest 0.1.1 [78], [79].

For time calibration, we used the divergence time between *Antirrhinum* and *Linaria* (13.33–27.32 Ma) from a previous estimate obtained in a relaxed molecular-clock analysis of tribe Antirrhineae (Vargas *et al.*, unpublished). This analysis was in turn calibrated with five Lamiales fossils and a divergence time between Oleaceae and Antirrhineae modeled as a normal distribution with mean = 74 Ma and Std = 2.5 Ma, on the basis of a relaxed molecular clock analysis of angiosperms [80], see [40] for details. We used the minimum age (13.33 Ma) as a fixed calibration point for the stem node of the *Linaria* clade to estimate the dates of the internal nodes with a penalized likelihood procedure implemented in r8s 1.71 [81]. Cross-validation to find the optimal smoothing parameter (10^k^) was done using increments of k of 0.1, from k = −3 to 3, repeated for two trees from the stable posterior distribution of each gene; the smoothing values of both trees were very similar so we used the value with lower χ^2^ error. After cross-validation we set the smoothing parameter to 1.5 for ITS, 3.2 for AGT1 and 0 for cpDNA and rate smoothed 20 trees drawn from the posterior distribution after burn-in to obtain the chronograms that were used in the coalescent simulations.

### Coalescent Simulations

We used simulations under the coalescent model following Maureira-Butler *et al*. [6] to test whether incomplete lineage sorting alone could explain the observed incongruence among gene trees. As the test does not account for the uncertainty of tree topology and branch length estimation, here we used 20 trees from the stable posterior distribution of the Bayesian analysis for each gene, performed the simulations and calculated all tree-to-tree distances from this pool of trees (hereafter the base line distribution), rather than the consensus as was done previously [6]. The base line distribution was then compared to the distribution obtained by calculating pairwise tree-to-tree distances of the 20 chronograms for each gene –essentially a measure of how much the gene trees from each locus differ– hereafter the observed distribution (see Methods S1 for further details).

Effective population size estimates (N_e_) used in the coalescent simulations were derived from cpDNA haplotypes and obtained via θ_w_ = 2µ*N_e,_* with theta (θ_w_) and mutation rate per generation (µ) taken from data of three *Linaria* species with contrasting range sizes (and potentially, contrasting N_e_) (table S2): *L. glacialis* (endangered, narrow endemic of Sierra Nevada, Spain), *L. elegans* (endemic to northern Iberia) and *L. simplex* (distributed across the Mediterranean basin). The effect of N_e_ estimates in the coalescent simulations was explored by repeating the set of simulations using the three N_e_ values separately (see Methods S1 for further details).

**Table 2:** Effect of taxa exclusion on the differences between base line (from simulated trees) and observed distributions of tree distances, numbers indicate steps while negative (-) and positive (+) values indicate approximation and separation between distributions, respectively.

	Effect after taxa deletion (steps)									
	ITS baseline distributionto ITS-cpDNA observeddistribution			AGT1 baseline distributionto AGT1-cpDNA observeddistribution			cpDNA baseline distributionto ITS-cpDNA observeddistribution			
Taxa with incongruentposition in gene trees	A	B	C	A	B	C	A	B	C	Averageeffect
*L. glauca* ssp. *olcadium* *	−4§	−2	−4	−3	−2	−2	−2	−2	−2	−*2.56*
*L. orbensis* *	−4	0	−4	+1	+1	+1	−2	−2	−2	−*1.22*
*L. amethystea* ssp. *amethystea* *	−3	−1	−3	−1	0	+1	−1	−1	−1	−*1.11*
*L. cuartanensis* *	+1	−1	−1	−1	−1	−1	−1	−2	−2	−*1.00*
*L. tursica* *	+2	+2	0	−2	−2	−1	−2	−2	0	−*0.56*
*L. oblongifolia* ssp. *oblongifolia* *	0	0	0	−2	−1	−1	0	0	0	−*0.44*
*L. alpina* *	0	+2	0	−1	−1	+1	0	−2	−2	−*0.33*
*L. filicaulis* *	0	0	0	−1	−1	−1	0	0	0	−*0.33*
*L. saturejoides* ssp. *saturejoides* *	0	0	0	−2	0	0	0	0	0	−*0.22*
*L. propinqua* *	−2	+1	−2	−1	+1	+1	0	0	0	−*0.22*
*L. supina* ssp. *supina*	0	+2	−2	−1	+1	+1	0	0	0	*+0.11*
*L. bubanii*	0	+2	−2	+1	−1	+1	0	−1	+1	*+0.11*
*L. bipunctata* ssp. *bipunctata*	−2	+2	0	−1	0	0	0	+2	0	*+0.11*
*L. saxatlis*	−2	+3	−2	+1	0	+1	0	0	0	*+0.11*
*L. badalii*	−1	+3	−1	−1	−1	−1	+1	+1	+1	*+0.11*
*L. almijarensis*	+1	+3	+1	−1	−1	−1	+1	0	0	*+0.33*
*L. mumbyana*	+1	+3	+1	−1	0	−1	+1	0	0	*+0.44*
*L. aeruginea*	+1	+1	+1	0	+1	+1	0	−1	0	*+0.44*

***A L. glacialis*** **N_e_:** Nuclear N_e_ = 190000, Plastid N_e_ = 95000.

***B L. elegans*** **N_e_:** Nuclear N_e_ = 320000, Plastid N_e_ = 160000.

***C L. simplex*** **N_e_,** Nuclear N_e_ = 680000, Plastid N_e_ = 340000.

* Individuals of putative hybrid origin that were excluded from the analysis in Figure 4.

**Figure 3:**
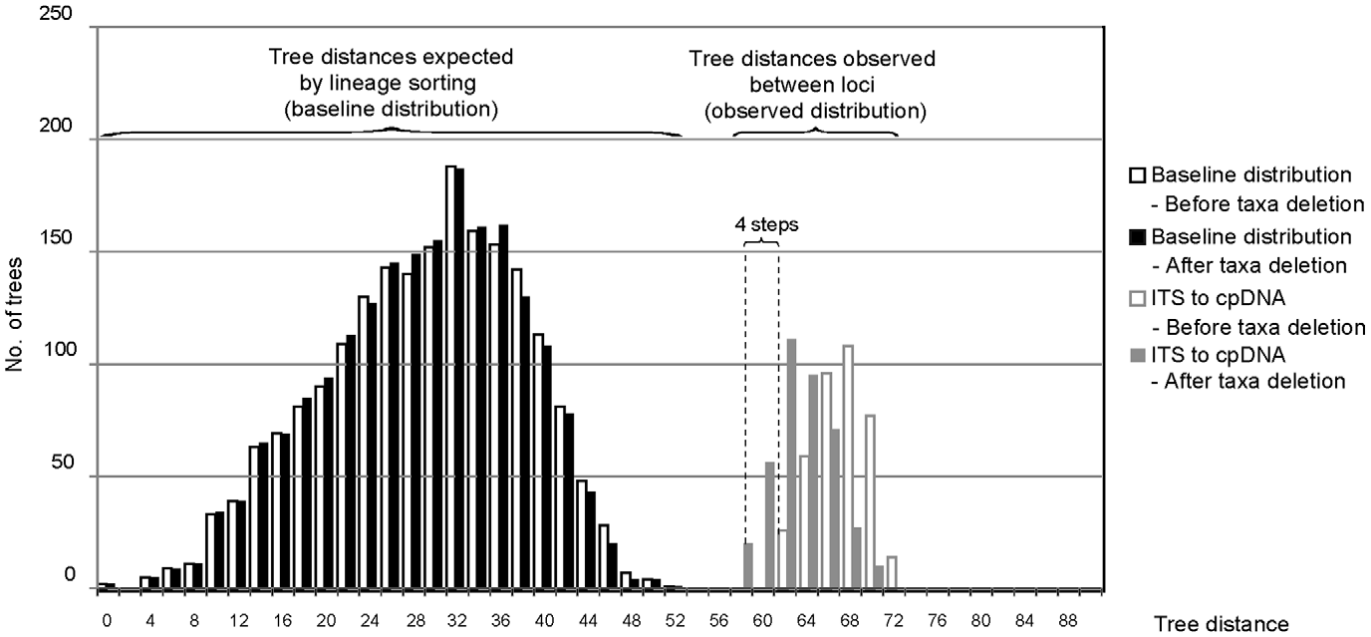
Detection of potential hybrids. An example illustrating the method used for the detection of potential hybrids. It is shown the effect of the exclusion of *L. glauca* ssp. *olcadium* on the differences between base line and observed distributions of tree distances.

§ Calculation plotted as an example in Fig. 3.

### Detection of Potential Hybrids

The detection of potential hybrids was addressed by examining the effect of taxon deletion on the observed and base line distributions. Theoretically, the potential hybrids detected by the test were the set of individuals that, after exclusion, retrieved overlapping observed distributions (pairwise tree-to-tree distances within their 95% HPD) and base line distributions (trees from coalescent simulations), thus the null hypothesis of incomplete lineage sorting alone was no longer rejected. Here, this approach was difficult to apply as the results were very dependent on the N_e_ values used (see Results). We identified that limitation, but we also recognized the significant challenge of getting exact estimates of population sizes through time in a phylogeny, especially with scarce genetic data [82], [83]. We then made an exploration of the effect of the deletion of each terminal with an incongruent position, in order to identify the individuals causing the highest effect in the differences between the baseline and the observed distributions. This was done by excluding terminals with incongruent positions (one at the time) and calculating new base line and observed distributions for the three datasets under each N_e_. The nine replications (three datasets x three N_e_) alleviated the non-reproducible effect of taxon exclusion due to the stochastic nature of simulations. The last step was to average the nine independent estimates obtained for each analyzed taxon.

### Testing Monophyly of *Supinae*

We used AGT1 and cpDNA datasets (one haplotype per sequence) with hybrids excluded to test support for the monophyly of *Supinae*. This was done to assess whether the incongruence (regarding *Supinae* naturalness) was exclusively explained by hybridization (as putative hybrids were excluded) and inference limitations, or whether additional processes generated real gene tree differences (in this case incomplete lineage sorting). In order to calculate support for the monophyly of *Supinae* we used two approaches: (i) the Shimodaira and Hasegawa [84] (S-H) test and the Bayes Factors [85], [86] (BF) test. The S-H test was implemented by calculating the maximum likelihood tree with unconstrained and constrained topologies in RAxML (–f d function) to subsequently compare both ML trees using the –f g function, which computes the per-site log Likelihoods for the contrasted topologies. The per-site log Likelihoods were analyzed with CONSEL [87] to obtain the S-H statistic values. BF test was used to assess alternative phylogenetic hypothesis in a Bayesian framework [85], [86]. The BF test quantifies the support for one hypothesis versus another given the data. We also used this approach, implemented in Tracer 1.4 [88] to test significant differences between the unconstrained and constrained Bayesian analyses of AGT1 and cpDNA. Stationarity and convergence of analyses were assessed in Tracer after discarding the first 10% of sampled generations as burn-in. Marginal likelihoods, their standard errors (estimated using 1000 bootstrap replicates) and BFs were calculated. We considered 2xlnBF(*H_1_* vs. *H*_0_) −2 to −6 as positive evidence against *H_1_ in favor of H*_0_; 2xlnBF(*H_1_* vs. *H*_0_) −6 to −10 as strong evidence against *H_1_ in favor of H*_0_; and 2xlnBF(*H_1_* vs. *H*_0_) <−10 as very strong evidence against *H_1_ in favor of H*_0_ [89].

**Table 3:** Results of Shimodaira-Hasegawa (S-H) test and Bayes Factors (BF) test with observed log-likelihood difference obtained in Maximum Likelihood analyses, S-H test statistics, mean values of marginal likelihood of the Bayesian analyses and BF test statistics (2xlnBF) for the unconstrained analysis (H_0_) and the analysis with monophyly of *Supinae* constrained in AGT1 and cpDNA datasets (H_1_).

Gene tree	Hypothesis (H)	S-H test		BF test	
		Observed log-likelihood difference	SH statistic	Marginal likelihood(lnP(model | data)) ± SE	2xlnBF(H vs. H0)
AGT1	H_0_	–	–	−3513.183±0.27	–
	H_1_: monophyly of sect. *Supinae*	35.9	0.01*	−3547.955±0.33	−69.546**
cpDNA	H_0_	–	–	−4461.125±0.27	–
	H_1_: monophyly of sect. *Supinae*	35.4	0.01*	−4488.746±0.26	−55.242**

* ≤0.05, support for rejection of H_1._

** ≤10, very strong evidence for rejection of H_1._

### Species Tree Inference

After excluding potential hybrids (to not violate the species tree model assumptions), we used the allelic data (and >1 individual per species in some cases, see Table S1) to estimate the species tree with the *BEAST (StarBeast) method [20] implemented in BEAST v.1.6.2. [89]. Allelic data were included in three data partitions with unlinked genealogies: (i) ITS sequences, (ii) AGT1 sequences and (iii) combined plastid (*rpl32-trnL^UAG^* and *trnS-trnG*) sequences. We used Sutton’s species delimitation [45], but additionally recognizing *L. almijarensis* Campo & Amo [64] (one population). The prior probability of the divergence time between *Linaria* and *Antirrhinum* was constrained to 20 Ma ±4 as a normal distribution, following date estimates obtained for the tribe Antirrhineae (Vargas *et al.*, unpublished, see “Gene trees estimation and calculation of dates” section). A Birth-Death process [90] was employed as the species tree branching prior. We used an uncorrelated lognormal relaxed clock model, with the prior probability for the substitution rate uniformly distributed, with ranges of 5×10^−4^-5×10^−2^ and 1×10^−4^-1×10^−2^ substitutions per site per Ma (s/s/Ma) for the nuclear loci and the plastid locus respectively. These rate constraints include previous estimates for herbaceous plant ITS rates (1.7–8.3×10^−3^ s/s/Ma) [91] and chloroplast rates (1.0–3.0×10^−3^ s/s/Ma) [92]. Nuclear synonymous substitution rates, being nearly neutral, may approximate nuclear intron rates. The former rates have been found in other plants to lie within the range we used (e.g., 48 *Gossypium* genes, 3.5–7.3×10^−3^ s/s/Ma, [93]; 39 legume genes, mean of 5.2×10^−3^ s/s/Ma, [94]). Six MCMC analyses were run for 30 million generations each, with a sample frequency of 1000. Analysis with Tracer v.1.5 [88] confirmed convergence of analyses and adequate sample sizes, with ESS values above 200. Analyses were combined using LogCombiner v.1.6.2 after discarding the first 10% generations of each run as burn-in. Trees were summarized in a maximum clade credibility tree using TreeAnotator v.1.6.2. After combination of the six log files from the analyses, the standard deviation of the uncorrelated lognormal relaxed clock (ucld.stdev) and the coefficient of variation (CoV) in the three genes were not close to 0: cpDNA ucld.stdev = 0.94, cpDNA Cov = 0.97; AGT1 ucld.stdev = 0.806, AGT1 CoV = 0.854; ITS ucld.stdev = 0.685, ITS CoV = 0.702. This branch rate heterogeneity indicated that the uncorrelated lognormal relaxed clock was appropriate.

**Figure 4:**
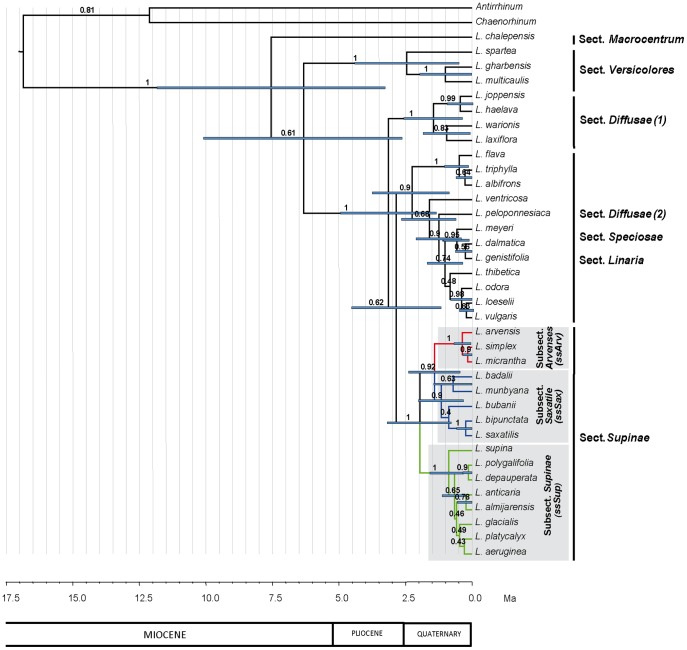
Species tree of *Linaria.* Maximum clade credibility tree obtained in the *BEAST species tree analysis after excluding potential hybrids and using allelic data of ITS, AGT1 and cpDNA datasets. Node bars represent the 95% highest posterior density intervals for the divergence time estimates of nodes with posterior probabilities above 0.50. Values above branches indicate Bayesian posterior probabilities. *Linaria* sections following Sutton (1988) are shown. Colors and clade labels represent the systematic nomenclature for *Supinae* as suggested in this paper.

**Table 4:** Morphological key traits of the subsections proposed for section *Supinae* regarding the results obtained in the *BEAST species tree analysis of ITS, AGT1 and cpDNA sequences (Figure 4).

	Subsect. *Arvenses*	Subsect. *Saxatile*	Subsect. *Supinae*
Corolla size	Small (2.5–9 mm)	Medium (6–18 mm)	Large (16–31 mm)
Seed wing shape	Thick-wide	Thick-wide/narrow (or absent)	Membranous-wide
Life-form	Annual	Annual/Perennial	Perennial

### Multilabelled Species Tree

A multilabelled species tree was inferred to retrieve the origin of the parental lineages of individuals affected by reticulation processes. We inferred a second species tree but this time including allelic data from potential hybrids. We recalculated the best-fitting model of sequence evolution with jModeltest 0.1.1 [78], [79], while the remaining priors were set as in the species tree analysis. The multilabelled species tree was built by assigning the two most congruent genes to one label (tip, or terminal species branch) and the remaining gene to a second label (see Table S3) while using missing data for the gene not assigned in the label. Thus, the two labels of a potential hybrid species (L1 and L2) where treated as different “species” in *BEAST analysis in order to show which two hybridizing lineages have contributed to a lineage of hybrid origin. The analysis therefore treated the differences between the two most congruent genes as being caused by incomplete lineage sorting alone, whereas our multilabelling approach allowed the differences between the most incongruent positions to be due to hybridization without violating the assumptions of the *BEAST model. The key concept is that a lineage of hybrid origin has two sources of parental contribution to its genome. These origins are best represented in a tree diagram by including two labels rather than just one (as is the case for lineages without a hybrid origin). This approach is novel, as far as we know, but has similarities to the approach used by Pirie *et al.* [95]. Four MCMC analyses were run for 100 million generations each, with a sample frequency of 10000. Analysis with Tracer v.1.5 [88] also confirmed convergence of analyses and adequate sample size, with ESS values above 200. We combined the analyses and summarized the tree as indicated above.

**Table 5:** Divergence dates of clades of *Linaria* sect *Supinae*, presented as mean crown ages and 95% highest posterior density (HPD) intervals based on the *BEAST species tree analysis (Figure 4).

Clade/Lineage	Mean age of divergence (Ma)	95% HPD interval
Genus *Linaria*	7.55	3.57–12.14
Sect. *Supinae*	1.97	0.87–3.28
Sect. *Supinae* subsect. *Arvenses*	0.36	0.08–0.72
Sect. *Supinae* subsect. *Saxatile*	1.16	0.39–2.08
Sect. *Supinae* subsect. *Supinae*	0.87	0.31–1.58

In order to contrast the results of the multilabelled species tree with other procedures widely used in phylogenetic studies, we also performed a *BEAST species tree analysis and a total evidence analysis, both with potential hybrids included.

## Results

### Haplotype Data

The Arlequin analysis gave us the two most probable haplotypes from the unphased genotypes of AGT1 and ITS sequences. For AGT1, we obtained haplotypes of 50 individuals with posterior probabilities (PP) above 0.95 and haplotypes of four individuals with PP below 0.95. For ITS, we obtained haplotypes of 34 individuals with PP above 0.95 and haplotypes of 20 individuals with PP below 0.95. The AGT1 phased data retrieved for the four individuals with low PP were empirically confirmed by amplicon cloning, recovering exactly the same allelic data that Arlequin inferred. As ITS is a multi-copy locus marker, there would be more than two copies for each unphased ITS genotype. This may have affected the haplotype detection, thus giving low support for the ITS haplotypes obtained. But (i) as one haplotype with low probability and differential position in the ITS allele-tree has been confirmed empirically (*L. bubanii*, 0.41 PP) and (ii) highly differentiated alleles have not been obtained in the Arlequin analyses (excluding *L. bubanii*), being all sister or closely-related in allelic-gene trees, we then considered that the two ITS haplotypes detected by Arlequin were good representatives of the existing ITS alleles per sample.

**Figure 5:**
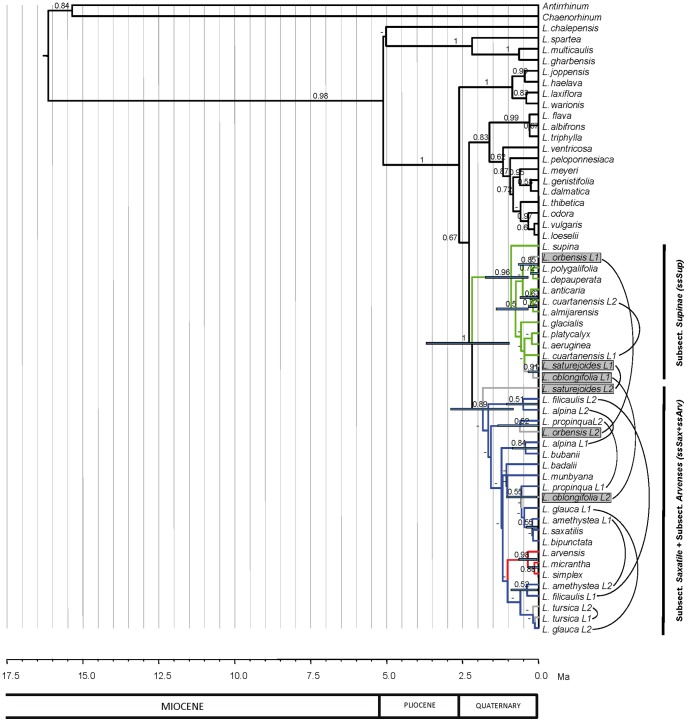
Reticulate evolution in *Supinae*. Maximum clade credibility tree obtained in the multilabelled *BEAST species tree analysis by including the presumed hybrids connected in two labels (L1 and L2) representing the two parental lineages of hybrid species. Node bars represent the 95% highest posterior density intervals for the divergence time estimates of nodes with posterior probabilities above 0.50 (only divergence time estimates for *Supinae* lineages are shown). Values above branches indicate Bayesian posterior probabilities. A hyphen (-) indicates posterior probability below 0.50. Colors and tree labels represent the systematic nomenclature for *Supinae* as established in this paper. Species labels of putative hybrids produced by the cross of the two main *Supinae* clades are highlighted in grey.

**Table 6:** Morphological key traits of species with putative hybrid origin produced by the cross between subsect *Saxatile* + subsect *Arvenses* (*ssSax+ssArv*) and subsect *Supinae* (*ssSup*) parental lineages based on the results obtained in the *BEAST multilabelled species tree analysis (Figure 5).

	*L. orbensis*	*L. saturejoides*	*L. oblongifolia*
Corolla size	Medium (11–15 mm)	Medium (12–17 mm)	Medium-large (15–22 mm)
Seed wing shape	Membranous-wide	Membranous-wide	Membranous-wide
Life-form	Annual	Annual	Annual

### Recombination Test

Recombination could not be detected in ITS by any of the five methods used. AGT1 showed one recombination event affecting several sequences that was detected by SiScan (Av. p-value = 3.712×10^−2^) but when contrasting the UPGMA trees of the recombinant and non-recombinant regions it showed almost the same topology with both potential parents separated in the tree from the potential recombinants. Additionally, evidence for recombination was not considered convincing if it only was detected by a single method as done in Poke *et al.* [96]. Therefore, we proceeded without removing the sequences under discussion.

**Table 7:** Divergence dates of parental lineages of hybrid species presented as mean age of divergence and 95% highest posterior density (HPD) intervals based on the *BEAST multilabelled species tree analysis (Figure 5).

Hybrid taxa	Mean age of divergencefrom most recent parentallineage (Ma)	95% HPDinterval	Mean age of divergence from 2^nd^ parentallineage (Ma)	95% HPD interval
*L. glauca ssp. olcadium*	0.61	0.17–1.17	1.53	0.62–2.68
*L. orbensis*	0.29	0.03–0.67	0.51	0.00–1.35
*L. amethystea ssp. amethystea*	0.17	0.02–0.38	0.36	0.00–0.89
*L. cuartanensis*	0.10	0.00–0.28	0.61	0.23–1.09
*L. tursica*	1.53	0.62–2.68	1.53	0.62–2.68
*L. oblongifolia ssp. oblongifolia*	0.12	0.00–0.36	0.61	0.17–1.17
*L. alpina*	0.35	0.00–0.86	0.35	0.00–1.04
*L. filicaulis*	0.35	0.00–1.04	0.36	0.00–0.89
*L. saturejoides* ssp. *saturejoides*	0.12	0.00–0.36	1.53	0.62–2.68
*L. propinqua*	0.51	0.00–1.35	0.61	0.17–1.17

### Gene Tree Inference

ITS phylogenetic analysis supported monophyly for section *Supinae* sister to a group formed by four species of sect. *Diffusae* (*L. laxiflora, L. warionis, L. haelava, L. joppensis*). In ITS, relationships within *Supinae* were not clearly related to morphological features (Fig. 1A). The AGT1 region did not support monophyly of the section, as species of sect. *Diffusae* and sect. *Versicolores* were grouped together with sect. *Supinae*. The three *Supinae* groups detected in AGT1 were also not obviously correlated with morphological characters (Fig. 1B). The cpDNA dataset did not support monophyly of the section, as there were two clearly separated groups of *Supinae* species, however, this locus showed three well-supported groups within *Supinae* associated with corolla sizes and seed shape (Fig. 1C).

**Table 8:** Previous phylogenetic studies of Mediterranean plants with highly supported incongruence among gene trees, we indicate those articles that claim hybridization and/or incomplete lineage sorting as major causes of topological inconsistency.

	Suggested cause of incongruence		
Mediterranean plant group	Hybridization	Incomplete lineage sorting	Reference
*Antirrhinum*	✓	✗	[102]
*Euphorbia* sect. *Aphyllis*	✓	✗	[110]
*Anthemis*	✓	✗	[111]
*Centaurium*	✓	✗	[112]
*Heliosperma*	✓	✗	[11]
*Reseda* sect. *Glaucoreseda*	✗	✓	[100]
*Ptilostemon*	✗	✓	[113]
*Hordeum*	✗	✓	[114]
*Amarillidaceae* (Mediterranean clade)	✗	✓	[115]
*Achillea*	✓	✓	[116]
*Senecio* sect. *Senecio*	✓	✓	[117]
*Arenaria* sect. *Plinthine*	✓	✓	[118]
*Phlomis crinita/lychnitis complex*	✓	✓	[119]
*Medicago*	✓	✓*	[6]

* Although not explicitly discussed, incongruence due to incomplete lineage sorting is also apparent among gene trees in this paper.

### Coalescent Simulations

When using the small and medium (*L. glacialis* and *L. elegans*, respectively) N_e_ estimates (Table S2), the pairwise distances of gene trees lay outside the base line distribution for either gene (Fig. 2). Contrastingly, when using the largest N_e_ values (from widespread *L. simplex*), the pairwise distances of gene trees lay inside the base line distribution of ITS and AGT1 genes (Fig. 2). As we expected a high overestimation of the population size when using *L. simplex* N_e,_ these results reflected that the degree of incongruence in the three gene trees was difficult to explain by incomplete lineage sorting alone when applying Maureira-Butler’s test [6].

### Detection of Potential Hybrids

When using simulations obtained with medium N_e_ values (*L.* *elegans*), only one individual needed to be removed in order to retrieve overlapping baseline and observed distributions (not shown), and therefore only one potential hybrid could be considered robustly detected. In contrast, when using simulations with the smallest N_e_ values (*L. glacialis*), even after removing all the individuals with incongruent positions, we still had non-overlapping distributions (not shown), and consequently all species with incongruent positions (17 spp.) were identified as potential hybrids. Therefore, our N_e_ estimates showed all possible scenarios: (i) gene tree incongruence is explained by incomplete lineage sorting alone (*L. simplex* N_e_), (ii) gene tree incongruence is explained by both incomplete lineage sorting and hybridization (*L. elegans* N_e_) and (iii) gene tree incongruence is explained by hybridization alone (*L. glacialis* N_e_). These results clearly illustrated the high dependence on N_e_ estimates in order to obtain the exact number of individuals of hybrid origin. We assumed that a reliable number of potential hybrids lay between the two extreme values obtained in (ii) and (iii).

The effect of the deletion of each incongruent individual on both the observed and base line distributions is shown in Table 2. We considered that individuals with the highest probability of hybrid origin were those individuals that, after deletion, decreased (on average) the differences between the base line and the observed distributions (in number of steps, see an example in Fig. 3). Ten of the 17 incongruent individuals decreased the differences among distributions and consequently were considered to be potential hybrids or to have a hybrid history in the broadest sense.

### Testing Monophyly of *Supinae*

After excluding putative hybrids, the S-H tests indicated that the constrained topologies for AGT1 and cpDNA had significantly worse likelihood scores than the unconstrained topologies (Table 3), thus monophyly of *Supinae* for these genes was statistically rejected. The BF test (Table 3) also recovered decisive (very strong) support (2xlnBF<−10) for rejection of monophyly of *Supinae* in the AGT1 and cpDNA. As monophyly of *Supinae* was recovered in ITS (Fig. 1A), topological incongruence in concert with S-H and BF test suggested that processes other than hybridization and inference limitations were also responsible for the topological incongruence among genes.

### Species Tree Inference

The *BEAST species tree analysis (potential hybrids excluded) (Fig. 4) retrieved four well supported groups within *Linaria*: (i) sect. *Versicolores* (1 PP), (ii) four species of sect. *Diffusae* (1 PP), (iii) a group formed by: three species of sect. *Diffusae*, four species of sect. *Speciosae* and five species of sect. *Linaria* (0.9 PP); and (iv) all sect. *Supinae* species (1 PP). Therefore sect. *Supinae* was retrieved as a monophyletic group with high support and was divided in three clades: one clade was represented by three annual species (*L. arvensis, L. simplex, L. micrantha*; 1 PP) with small corollas (2.5–9 mm) and a thick-wide seed wing (subsect. *Arvenses,* hereafter *ssArv*). A second clade was represented by five annual or perennial species (*L. badalii, L. munbyana, L. bubanii, L. bipunctata, L. saxatilis*; 0.90 PP) with medium-sized corollas (6–18 mm) and a thick-wide seed wing or narrow wing (marginal ridge) (subsect. *Saxatile,* hereafter *ssSax*). The third clade contained eight perennial species (*L. supina, L. polygalifolia, L. depauperata, L. anticaria, L. almijarensis, L. glacialis, L. platycalyx, L. aeruginea*; 1 PP) with large corollas (16–31 mm) and a membranous-wide seed wing (subsect *Supinae,* hereafter *ssSup*) (see Table 4).

The * BEAST species tree detected that incomplete lineage sorting has affected all gene trees analyzed. In the ITS dataset we detected deep coalescence at medium depth branches (see *L. bubanii* position in the ITS tree and *BEAST species tree); from the AGT1 dataset we detected deep coalescence at medium depth branches (*L. munbyana, L. badalii*) and at deeper branches (*L. polygalifolia, L. depauperata, L. orbensis, L. anticaria, L. almijarensis, L. aeruginea, L.glacialis* and *L. platycayx*); in cpDNA we also detected deep coalescence at the deepest branches (*L. badalii, L. bubanii, L. munbyana, L. bipuncata* and *L. saxatilis*).

The time to the most recent common ancestor (TMRCA) of *Supinae* was placed in the late Pliocene-early Pleistocene (0.87–3.28 Ma), the TMRCA of *ssArv* was located in the middle-late Pleistocene (Ionian-Tarantian) (0.08–0.72 Ma), the TMRCA of *ssSax* in the early-middle Pleistocene (Gelasian-Calabrian-Ionian) (0.39–2.08 Ma) and the TMRCA of *ssSup* in the early-middle Pleistocene (Calabrian-Ionian) (0.31–1.58 Ma) (see Table 5).

### Multilabelled Species Tree

The multilabelled species tree (Fig. 5) retrieved a well supported clade (0.96 PP, *ssSup*) and a clade with moderate support (0.89 PP, *ssSax+ssArv*) within *Supinae*. Out of ten reticulation events that have been presumed to occur, one was produced within the *ssSup* clade, six within the *ssSax+ssArv* clade and three between these two clades. One of the six potential hybridization events within ssSax+ssArv clade is reflected in *L. tursica*, a species with morphological traits typical from both ssSax and ssArv clades (Fig. 4): wingless seed (some species of ssSax present narrow to marginal seed wings) and small corolla (ssArv). The three reticulation events inferred between ssSup and ssSax+ssArv produced three species with morphological traits typical of both clades (*L. orbensis, L. saturejoides and L. oblongifolia*, see Fig. 5 and Table 6).

We estimated the timing of the hybridization events by looking at the divergence time of parental lineages of putative hybrids. As hybridization could not take place prior to divergence of parental lineages, divergence time for the most recent lineage constituted the maximum age of each hybridization event. Despite the topological uncertainty at the tips, we found that all bar one maximum age of the presumed hybridization episodes occurred during the Pleistocene (Fig. 5 and Table 7). In a single case, *L. tursica*, the 95% HPD overlapped the Middle Pliocene, although the mean estimate remained within the Pleistocene (Table 7).

## Discussion

### Using a Coalescent Framework to Disclose the Evolutionary History of *Supinae*

Systematics of *Linaria* and specifically sect. *Supinae* has been subject to various interpretations in numerous taxonomic treatments in the last two centuries. Historical disagreement occurred when discerning the naturalness of the section and its internal classification [43]–[51] (see Table 1). To disclose the evolutionary history of *Supinae*, we sampled genetic data from 46 *Linaria* species, including sequences from three presumably unlinked genes. Because of the highly supported incongruence among trees based on separate analysis of the three genes, difficulty in the systematic reconstruction of *Supinae* at this stage of analysis was patent, the naturalness of the section remained unclear and the infra-sectional classification was still controversial.

In the last few years the incorporation of the coalescent model into phylogenetic analysis has greatly improved the theoretical basis for inferring species trees from gene trees via a mixed model –the multispecies coalescent (e.g., BEST [28]; *BEAST [20]; [97]). One key practical challenge is to include only data that meet the assumptions of the current implementations. Of significant concern is to properly handle sequences, individuals or taxa with multiple histories, such as by excluding recombinants or hybrids prior to species tree inference.

Here, we performed simulations under coalescence following the method of Maureira-Butler *et al.* [6] to estimate whether the gene tree incongruence detected among genes could be explained by incomplete lineage sorting without hybridization. The test exposed that with small and medium N_e_ values used in the simulations, the topological variation generated by incomplete lineage sorting was not as high as the incongruence observed between the three genes (Fig. 2), whereas with high N_e_ (*L. simplex* N_e_)_,_ the variation generated by incomplete lineage sorting alone could explain the totality of incongruence observed between genes (Fig. 2). We considered that the high N_e_ greatly overestimated the general N_e_ of *Linaria*_,_ as only 9 out of 150 *Linaria* species have a similar wide range size [45] (and presumably similar N_e_). Hence, the results of Maureira-Butler’s test suggested that incongruence among genes was difficult to explain by incomplete lineage sorting alone, indicating that hybridization may also account for the gene tree inconsistency. However, the exact number and identity of individuals that may have hybrid histories is not clearly established here, because of the sensitivity of the test to N_e_ estimation. We consider, instead, that the test has provided a probable set of individuals that may adversely affect the *BEAST analysis and that a cautious approach (removing these individuals before the analysis) is preferred here, rather than risking a spurious species tree inference.

The hybrid detection test (Table 2) and the multilabelled *BEAST species tree (Fig 5) was also contrasted with a *BEAST species tree including all potential hybrids (not shown). After six runs with 30 million generations, convergence could not be reached and some ESS values (of population size parameters) remained under 200, which illustrated that the inclusion of potential hybrids may be violating assumptions of the *BEAST analysis. Our approach was also contrasted with an additional analysis of the three datasets concatenated in a total evidence approach (see Fig. S1). Results of both approaches (our multilabelled species tree with hybrids excluded vs. the total evidence analysis) gave highly conflicting results. These discordant results were expected, as it is known that concatenation of data from multiple loci may lead to biased phylogenetic estimates under widespread incomplete lineage sorting and/or hybridization [19]. Results presented here highlight the paramount importance of (i) analyzing multiple loci datasets in a multispecies coalescent approach in order to find a more realistic species tree and (ii) the requirement of additional analytical tools to identify and to disclose the origin of species affected by historical hybridization. We note that our multilabelled species tree still allows the possibility of observing congruent placements for each label of the same individual. That is, we are not forcing different placements with this approach, but instead allowing them, if preferred by the data. Therefore, this approach appears to combine the ideals of utilizing the available comparable data sets (including hybrids) while also appropriately accommodating processes that may cause incongruence (incomplete lineage sorting) and could otherwise lead to spurious tree inference.

### Systematics and Drivers of Evolution in *Supinae*

The *Linaria* *BEAST species tree retrieved three well supported clades that agreed with previous classifications (Fig 4): (i) Sect. *Versicolores*, (ii) four species of Sect. *Diffusae* and (iii) Sect. *Supinae.* It also retrieved a group that was incongruent with previous taxonomic treatments. This latter group contained three species of Sect. *Diffusae*, four species of Sect. *Speciosae* and five species of Sect. *Linaria*. In this analysis *Supinae* was monophyletic, as found in the ITS phylogeny. Furthermore, *Supinae* was divided into three morphologically-based subclades consistent with life-form, corolla size and seed wing shape (Table 4), as found in the cpDNA phylogeny: subsect. *Supinae* (*ssSup*), subsect. *Arvenses* (*ssArv*) and subsect. *Saxatile* (*ssSax*). These results are strikingly consistent with some earlier hypotheses, despite the incongruence observed among gene trees. *ssSup* contained eight species that were grouped together in several previous morphological classifications, *ssArv* contained three species that were also previously grouped in a taxonomical entity, whereas *ssSax* contained five species that were historically placed in several distinct taxonomic groups (Systematic proposal in Table 1, diagnostic characters in Table 4). Corolla size and seed wing shape were also previously used as diagnostic characters in a morphological taxonomic revision of winged-seeded *Linaria* species [46]. This author considered *Arvenses (ssArv)* (small flowers) as an independent section and divided *Supinae* in three subsections according to life form and seed wing shape: (i) subsect. *Supinae (ssSup)*: perennial plants with membranous seed wings, (ii) subsect. *Amethystea*: annual plants with thick seed wings and (iii) subsect. *Saxatile:* annual or perennial plants with somewhat thin wings.

Reproductive biology and interaction with pollinators may have played an important role in differentiation within *Supinae*. This is supported by the fact that the species with very low investment in flower structures (small corollas, *ssArv*) are all self-compatible, whereas species with a high investment in flower formation (large corollas, *ssSup*) are all self-incompatible, mainly pollinated by large bees and with low pollinator diversity (Blanco-Pastor & Vargas, unpublished). Geography appears to have played a role in structuring the diversity within *Supinae* as the diversity of *ssSax* is located in the northern part of the Iberian Peninsula (three out of the five species are northern Iberian endemics), whereas the diversity of *ssSup* is located in southern Iberia (five out of eight species are southern Iberian endemics). The timing of divergence of the three subclades (crown nodes, Table 5) indicates that diversification occurred during the Quaternary, after the establishment of the Mediterranean climate regime [39], when species had to tolerate the climatic oscillations occurring in that period [98], [99]. This pattern of geographical differentiation driven by Quaternary interglacial fragmentation has been previously identified in many Iberian plants [36], [37], [100], including the closely-related genus *Antirrhinum* [101], [102].

### Hybridization during the Quaternary Glaciations

We found that historical hybridization has been likely during the course of *Supinae* evolution. Our analyses identified 10 out of 17 individuals with incongruent positions in gene trees that were difficult to reconcile with incomplete lineage sorting (Table 2). Simple introgression (that is, recurrent horizontal gene flow toward one parental species without formation of new species) can explain the observed gene tree incongruence in those individuals. But the observed pattern could have been also generated by homoploid hybrid speciation (all *Linaria* species analyzed here are diploid (2n = 12) excluding *L. chalepensis* (2n = 24)). Despite speciation via homoploid hybridization has been historically hard to detect (as it could present a similar signal to simple introgression or incomplete lineage sorting) [31], recent studies have suggested that it might be an important mechanism for plant speciation [58], [103], [104]. Our analyses do not validate speciation via homoploid hybridization, but this process must not be discarded as potential generator of diversity in *Supinae*.

The multilabelled *BEAST species tree analysis (Fig. 5) recovered, to some degree, the origin of the parental alleles of individuals affected by historical hybridization. There is bound to be a loss of power, because of the reduced number of loci available to place the multilabelled species as well as the need to use missing data. Even so, out of ten potential hybridization events detected, our analyses suggested that one occurred within the *ssSup* lineage, three between two distant parental lineages (*ssSax+ssArv* and *ssSup*) and six within the *ssSax+ssArv* lineage. Crosses between the two distant parental lineages retrieved in the analysis (*ssSax+ssArv* and *ssSup*) were also supported by morphology, given that those three taxa (*L. orbensis, L. oblongifolia* and *L. saturejoides*) presented morphological key traits from both clades (Table 6). All hybridization events inferred here were also supported by the results obtained in experimental crosses performed by Valdés [53]. In that study, this author obtained fruits in one of the four crosses performed among *ssSup* species, three of the four crosses between *ssSax+ssArv* and *ssSup* species and four of the seven crosses among *ssSax+ssArv* species (note that here we only accounted for crosses produced between species used in this study thus a higher number of total successful crosses were produced, see [53]).

The maximum age of a hybridization event was considered here to be the maximum age of the origin of the most recent parental lineage. Those ages were circumscribed between 0.28–1.35 Ma in nine of the ten potential hybrids (Table 7). Only in *L. tursica* did the maximum age of hybridization surpass 2.5 Ma (2.68 Ma). Taking into account the effect of low phylogenetic resolution that obscured the detection of ages in parental lineages (thus considering the maximum age of hybridization at deeper nodes), the present results lead us to affirm that all potential hybridization events detected but one may have occurred during the Pleistocene climatic oscillations. During the Quaternary, hybrid zones were established in contact zones (Pyrenees, Alps, Central Europe and Scandinavia) of interglacial northward colonization routes from the temperate regions of Europe [105], [106]. In the Iberian Peninsula, where ice effects were less severe, subsequent patterns of contraction, fragmentation, persistence, expansion and admixture during altitudinal migrations may have repeatedly produced multiple hybrid zones [33], [34], [99]. The complex Iberian orography may have allowed partial differentiation of lineages in allopatry but subsequent secondary contacts of differentiated genomes from close locations [34]. That may have been the framework for *Linaria* and many other southern European plant groups (Table 8). Clearly, the investigation of hybridization in Mediterranean plant groups is vital for the accurate inference of species trees, as well as to understand the role of hybridization in the generation of new genetic combinations and morphological differentiation. However, we have shown in this example that existing tools, although limited, can nonetheless provide valuable insights in these areas.

### Incomplete Lineage Sorting as a Significant Process in Mediterranean Plants

Several studies have claimed incomplete lineage sorting as a major cause of gene tree incongruence and non-monophyly in Mediterranean plants (Table 8). Failure of gene lineages to coalesce occurs when the time between speciation events is very short and/or when the effective population size of the ancestral populations is very large [2]. We detected incomplete lineage sorting in all independent loci analyzed for *Linaria*. In this genus, population size estimates obtained by using three *Linaria* species (*L. glacialis, L. elegans, L. simplex*) suggested that ancestral populations may have not been extremely large (see [107] for comparison). Conversely, extremely rapid divergence of ancestral populations seems more likely. *Linaria* has diversified since the late Miocene-early Pliocene (3.57–12.14 Ma) (crown node of the genus, Table 5) to recent times in the late Quaternary (Table 5, Fig. 4). During its evolutionary history, this Mediterranean group may have experienced drastic climatic events such as the Messinian Salinity Crisis (5.96 Ma) [108], the catastrophic flood that caused the refilling of the Mediterranean Sea (5.33 Ma) [109], the progressive establishment of the Mediterranean rhythm with dry summers (3.2 Ma) and the Quaternary type oscillations with glacial and interglacial stages (2.3 Ma) [39]. These extreme climatic changes coupled with the irregular mountain ranges of the Mediterranean basin might have promoted rapid diversification driven by isolation in reduced areas causing rapid allopatric speciation. The secondary contacts occurring during the climatic oscillations seem to have promoted historical hybridization between closely related *Linaria* species, but also the high number of species in the Mediterranean (104 spp.) [45] and its recent origin suggest that this group is likely to have undergone rapid diversification. Additional analyses not performed here are proposed to confirm rapid speciation as the cause for incomplete lineage sorting in *Linaria*.

The basis underlying phylogenetic incongruence may vary depending on the plant group under study, but the flora of the Mediterranean is formed, in part, by many genera that similarly display numerous species generated in short periods of time that also may have suffered secondary contacts in short term cycles (20.000–100.000 yr.). In these groups incomplete lineage sorting and hybridization appear to be the rule rather than the exception.

## Supporting Information

Figure S1 **Total evidence analysis.** The 50% majority-rule consensus tree obtained in the Bayesian analysis of the concatenated ITS, AGT1 and cpDNA datasets. Numbers above branches are Bayesian posterior probabilities. Colors represent the systematic nomenclature for *Supinae* as suggested in this paper (see Fig. 4). Species with intermediate key traits are represented in grey.
(TIF)

Table S1 **List of taxa included with localities, collector’s numbers and Genbank accession numbers.**
(DOCX)

Table S2 **Effective population size estimates (N_e_) used in the coalescent simulations.**
(DOCX)

Table S3 **Assignment of genes to label 1 (L1) or label 2 (L2) in the multilabelled species tree analysis (** **Fig. 5** **).**
(DOCX)

Methods S1 **Supplemental methods.**
(DOCX)
