# Supplementary figures and images for: Coalescent Simulations Reveal Hybridization and Incomplete Lineage Sorting in Mediterranean Linaria
Source: PLoS One. 2012 Jun 29;7(6):e39089. doi: 10.1371/journal.pone.0039089 (PMC3387178; doi:10.1371/journal.pone.0039089)

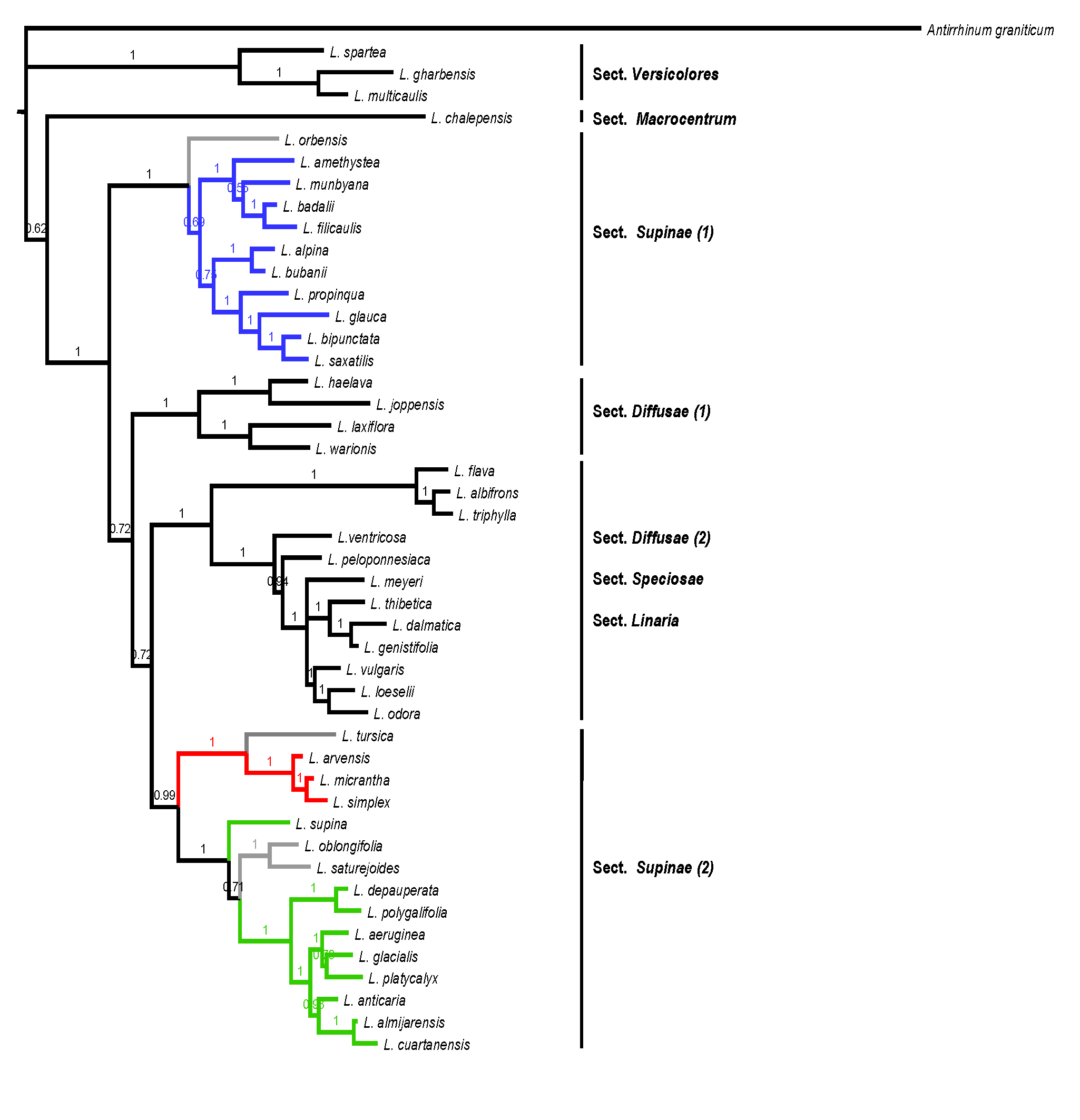

Supplement: Figure S1 — Total evidence analysis. The 50% majority-rule consensus tree obtained in the Bayesian analysis of the concatenated ITS, AGT1 and cpDNA datasets. Numbers above branches are Bayesian posterior probabilities. Colors represent the systematic nomenclature for Supinae as suggested in this paper (see Fig. 4). Species with intermediate key traits are represented in grey. (TIF) [file pone.0039089.s001.tif]
