# Supplementary material for: Coalescent Simulations Reveal Hybridization and Incomplete Lineage Sorting in Mediterranean Linaria
Source: PLoS One. 2012 Jun 29;7(6):e39089. doi: 10.1371/journal.pone.0039089 (PMC3387178; doi:10.1371/journal.pone.0039089)
Supplement: Table S1 — List of taxa included with localities, collector’s numbers and Genbank accession numbers. (DOCX) [file pone.0039089.s002.docx]

**Table S1.** List of taxa included in the present paper with localities, collector’s numbers and Genbank accession numbers.

| **Taxon** | **Locality** | **Collector** | **Collection number (herbarium)** | **ITS** | ***trnS-trnG*** | ***rpl32-trnL^UAG^*** | ***Agt1*** |
| --- | --- | --- | --- | --- | --- | --- | --- |
| **Sect. *Supinae*** |  |  |  |  |  |  |  |
| *L. aeruginea* (Gouan) Cav. ssp. *aeruginea* | Spain: Granada. Sierra Nevada. Pradollano | J.L. Blanco Pastor | 51JB09 | JQ814486 | JN663616 | JN663397 | JQ814558 |
| *L. aeruginea* ssp*. nevadensis* (Boiss.) D.A. Sutton | Spain: Granada. Sierra Nevada. Pico del Veleta | J.L. Blanco Pastor | 44JB09 | JQ814487 | JN663620 | JN663401 | JQ814559 |
| *L. almijarensis* Campo & Amo | Spain: Córdoba. Cabra. | J.L. Blanco Pastor | 36JB10 | JQ814492 | JN663632 | JN663412 | JQ814564 |
| *L. alpina* (L.) Mill. | Spain: Huesca. Formigal | S. Martín Bravo | 571SMB05 | JQ814489 | JN663622 | JN663510 | JQ814561 |
| *L. amethystea* (Vent.) Hoffmanns. & Link *ssp. amethystea* | Spain: Ciudad Real. Ciudad Real | R. García Río | 712742 (MA) | JQ814490 | JN663624 | JN663405 | JQ814562 |
| *L. anticaria* Boiss. & Reut. | Spain: Málaga. El Torcal de Antequera | J.L. Blanco Pastor | 33JB09 | JQ814491 | JN663628 | JN663408 | JQ814563 |
| *L. arvensis* (L.) Desf. (1) | France: Córse. Col de Bigorno. | J. Lambinon | 2009.12.169 (RNG) | JQ814493 | JN663635 | JN663415 | JQ814565 |
| *L. arvensis* (L.) Desf. (2) | Spain: Almería. Uleila del Campo | S.L. Jury & R.N. Carter | 2009.12.165 (RNG) | JQ814494 | JN663636 | JN663416 | JQ814566 |
| *L. badalii* Loscos | Spain: León. Riaño | M.F. Gardner & S.G. Gardner | 2009.12.155 (RNG) | JQ814495 | JN663641 | JN663421 | JQ814567 |
| *L. bipunctata* (L.) Chaz. ssp. *bipunctata* | Spain: Soria. Quintana Redonda | A. Segura | 2009.12.7 (RNG) | JQ814496 | JN663643 | JN663423 | JQ814568 |
| *L. bubanii* Font Quer | Spain: Huesca. El Pueyo de Araguas | M. Carrasco & al. | 609430 (MA) | JQ814537 | JN663644 | JN663424 | JQ814569 |
|  |  |  |  | JQ814538 |  |  |  |
| *L. cuartanensis* (Degen & Hervier) Fern. Casas ex. Blanco-Pastor | Spain: Albacete. Yeste | P. F. Cannon et al. | 2009.12.35 (RNG) | JQ814510 | JN663667 | JN663444 | JQ814583 |
| *L. depauperata* Leresche ex Lange ssp. *depauperata* | Spain: Alicante. Alcoi | L. Serra | 2009.12.157 (RNG) | JQ814499 | JN663651 | JN663430 | JQ814572 |
| *L. filicaulis* Boiss. ex Leresche & Levier | Spain: León. Pico Tres Provincias | C.M. Romero Rodríguez | 769084 (MA) | JQ814500 | JN663655 | JN663434 | JQ814573 |
| *L. glacialis* Boiss. (1) | Spain: Granada. Sierra Nevada. Corral del Veleta | J.L. Blanco Pastor | 43JB09 | JQ814504 | JN663659 | JN663437 | JQ814577 |
| *L. glacialis* Boiss. (2) | Spain: Granada. Sierra Nevada. El Caballo. | J.L. Blanco Pastor | 70JB09 | JQ814505 | JN663660 | JN663438 | JQ814578 |
| *L. glauca ssp. olcadium* Valdés & D.A. Webb | Spain: Albacete. Balazote | Rivas Goday | 2009.12.151 (RNG) | JQ814506 | JN663663 | JN663441 | JQ814579 |
| *L. micrantha* (Cav.) Hoffmanns. & Link. | Spain: Huelva. Marismas del Odiel | J.L. Blanco Pastor | 22JB09 | JQ814513 | JN663669 | JN663446 | JQ814585 |
| *L. munbyana* Boiss. & Reut. | Spain: Huelva. Marismas del Odiel | J.L. Blanco Pastor | 21JB09 | JQ814515 | JN663670 | JN663447 | JQ814587 |
| *L. oblongifolia* (Boiss.) Boiss. & Reut. *ssp. oblongifolia* | Spain: Málaga. El Torcal de Antequera | J.L. Blanco Pastor | 34JB09 | JQ814516 | JN663672 | JN663449 | JQ814588 |
| *L. orbensis* Carretero & Boira. | Spain: Alicante. Sagra | J.L. Blanco Pastor | 4JB10 | JQ814518 | JN663676 | JN663453 | JQ814590 |
| *L. platycalyx* Boiss. | Spain: Cádiz. Zahara de la Sierra | S. Martín Bravo | 5SMB08 | JQ814520 | JN663677 | JN663454 | JQ814592 |
| *L. polygalifolia ssp. lamarckii* (Rouy) D.A. Sutton. (1) | Portugal: Algarve. Monte Gordo | J.L. Blanco Pastor | 33JB10 | JQ814522 | JN663679 | JN663456 | JQ814594 |
| *L. polygalifolia ssp. lamarckii* (Rouy) D.A. Sutton. (2) | Spain: Huelva. Isla Canela. | J.L. Blanco Pastor | 19JB09 | JQ814521 | JQ814550 | JQ814617 | JQ814593 |
| *L. polygalifolia* Hoffmanns. & Link *ssp. polygalifolia* | Portugal: Estremadura. Guincho | H.J.M. Bowen | 2009.12.108 (RNG) | JQ814523 | JN663680 | JN663457 | JQ814479 |
|  |  |  |  |  |  |  | JQ814483 |
| *L. propinqua* Boiss. & Reut. | Spain: Bilbao. Zeanurri | J.A. Alejandre | 468162 (MA) | JQ814524 | JN663682 | JN663459 | JQ814595 |
| *L. saturejoides* Boiss. ssp. *saturejoides* | Spain: Málaga. Sierra Tejeda. Canillas de Aceituno | J.L. Blanco Pastor | 36JB09 | JQ814525 | JN663685 | JN663462 | JQ814596 |
| *L. saxatilis* (L.) Chaz. | Spain: Ávila. Hoyos del Espino | P. Vargas | 94PV09 | JQ814526 | JN663687 | JN663464 | JQ814597 |
| *L. simplex* Willd. ex Desf. (1) | Greece: Ararchova | P. Vargas | 79PV08 | JQ814528 | JN663697 | JN663474 | JQ814599 |
| *L. simplex* Willd. ex Desf. (2) | Spain: Granada. Orjiva | R.N. Carter | 2009.12.162 (RNG) | JQ814527 | JN663699 | JN663476 | JQ814598 |
| *L. supina* (L.) Chaz. *ssp. supina* | France: Gorges de l’Hérault | J. Lambinon | 2009.12.131 (RNG) | JQ814530 | JN663704 | JN663481 | JQ814601 |
| *L. tursica* Valdés & Cabezudo. | Spain: Huelva. Coto de Doñana | J.L. Blanco Pastor | 18JB09 | JQ814533 | JN663715 | JN663492 | JQ814603 |
| ***Other sections*** |  |  |  |  |  |  |  |
| *L. albifrons* (Sibth. & Sm.) Steudel (Sect. Diffusae) | Israel. Negev. Beer Sheva | A. Danin, S.G. Knees et al. | 2010/12/07 (RNG) | JQ814488 | JQ814539 | JQ814606 | JQ814560 |
| *L. chalepensis* (L.) Mill. (Sect. Macrocentrum) | Cyprus: Larnaca, Cape Kiti | Iter Mediterraneum IV | 495681(MA) | JQ814497 | JN663647 | JF694128 | JQ814570 |
| *L. dalmatica* (L.) Miller (Sect. Speciossae) | Bulgaria: Central Rhodopes | C. Navarro & al. | 726987 (MA) | JQ814498 | JQ814540 | JQ814607 | JQ814571 |
| *L. flava* (Poiret) Desf. (Sect. Diffusae) | Italy: Corsica. Camp dell'Oro. Ajaccio | C. Bukanell & L. Ollum | 00419551 (E) | JQ814501 | JQ814541 | JQ814608 | JQ814574 |
| *L. genistifolia* (L.) Miller (Sect. Speciosae) | Turkey: Hadim-Bezkir | JJ. Aldasoro & M.L. Alarcón | A9751 | JQ814502 | JQ814542 | JQ814609 | JQ814575 |
| *L. gharbensis* Batt. & Pit. (Sect. Versicolores) | Spain: Huelva. Gibraleón | M. Fernández-Mazuecos | 7MF09 | JQ814503 | JN663658 | JF694139 | JQ814576 |
| *L. haelava* (Forskål) F.G. Dietr. (Sect. Diffusae) | Israel: Horbat Medin | D. Heller & I. Shammash | 532177 (MA) | JQ814507 | JQ814543 | JQ814610 | JQ814580 |
| *L. joppensis* Bornm. (Sect. Diffusae) | Israel: Philistean plain. Ashkeleton | A. Danin, S.G. Knees et al. | 2010/12/11 (RNG) | JQ814508 | JQ814544 | JQ814611 | JQ814581 |
| *L. laxiflora* Desf. (Sect. Diffusae) | Tunisia: Jerid. Cedada. Sidi Ben Arbessidi Bouhlel | C. Aedo & al. | 795183 (MA) | JQ814509 | JQ814545 | JQ814612 | JQ814582 |
| *L. loeselii* Schweiger (Sect. Linaria) | Lithuania. Apskritis ol Klaipeda. Curonian Spit. | E. Glazkova & A. Quintanar | 791644 (MA) | JQ814511 | JQ814546 | JQ814613 | JQ814584 |
| *L.meyeri Kuprian* (Sect. Linaria) | Georgia: Mtskhete Mtianeti. Gran Caucaso | L. Muñoz & al. | 764400 (MA) | JQ814512 | JQ814547 | JQ814614 | JQ814476 |
|  |  |  |  |  |  |  | JQ814480 |
| *L. multicaulis* (L.) Mill. (Sect. Versicolores) | Morocco: Azrou | M. Fernández-Mazuecos | 15MF08 | JQ814514 | JN663719 | JF694155 | JQ814586 |
| *L. odora* (Bieb.) Fisher (Sect. Linaria) | Russia. Voilgograd. Frolavo. Prov. Gulajerka | A.K. Skvortsov | 00419545 (E) | JQ814517 | JQ814548 | JQ814615 | JQ814589 |
| *L. peloponnesiaca* Boiss. & Heldr. (Sect. Speciosae) | Greece: Mt. Olympus | P. Vargas | 778352 (MA) | JQ814519 | JQ814549 | JQ814616 | JQ814591 |
| *L. spartea* (L.) Chaz. (Sect. Versicolores) | Spain: Cáceres. Monfragüe | M. Fernández-Mazuecos | 4MF08 | JQ814529 | JN663701 | JN663478 | JQ814600 |
| *L. thibetica* Franchet (Sect. Linaria) | China: Sichuan prov. Siangcheng Xian. between Xiangcheng and Daxue | D.E. Boufford & al. | 00292244 (E) | JQ814531 | JQ814551 | JQ814618 | JQ814602 |
| *L. triphylla* (L.) Miller (Sect. Diffusae) | Tunisia: El Vef. between Sidi Merzong and Aïn Linghassel | J. Calvo & al. | 797461 (MA) | JQ814532 | JQ814552 | JQ814619 | JQ814478 |
|  |  |  |  |  |  |  | JQ814482 |
| *L. ventricosa* Cosson & Bal. (Sect. Speciosae) | Morocco: Meknès-Tafilalet | T. Buira, J. Calvo & S. Hartson | 807960 (MA) | JQ814534 | JQ814553 | JQ814620 | JQ814604 |
| *L. vulgaris* Miller (Sect. Linaria) | France: Chamonix | B. Estébanez | s.n. | JQ814535 | JQ814554 | JQ814621 | JQ814477 |
|  |  |  |  |  |  |  | JQ814481 |
| *L.warionis* Pomel (Sect. Diffusae) | Morocco: Beni Tajjita, route vers Talsinat | D.Podlech | 589733 (MA) | JQ814536 | JQ814555 | JQ814622 | JQ814605 |
| ***Outgroup*** |  |  |  |  |  |  |  |
| *Antirrhinum graniticum* Rothm. | Spain: Cáceres. Trujillo | P. Vargas | 213PV06 | JQ814484 | JN663609 | JF694120 | JQ814556 |
| *Chaenorhinum.macropodum* (Boiss. & Reut.) Lange | Spain: Málaga. Cómpeta | M. Fernández-Mazuecos | 7E3MF08 | JQ814485 | JN663610 | JF694119 | JQ814557 |
