# Supplementary material for: Coalescent Simulations Reveal Hybridization and Incomplete Lineage Sorting in Mediterranean Linaria
Source: PLoS One. 2012 Jun 29;7(6):e39089. doi: 10.1371/journal.pone.0039089 (PMC3387178; doi:10.1371/journal.pone.0039089)
Supplement: Table S2 — Effective population size estimates (Ne) used in the coalescent simulations. (DOCX) [file pone.0039089.s003.docx]

**Table S2.** Effective population size estimates (N_e_) used in the coalescent simulations.

|  | Simulations from ITS and AGT1trees | Simulations from cpDNA trees |
| --- | --- | --- |
| *L. elegans* | 320 000 | 160 000 |
| *L. glacialis* | 190 000 | 95 000 |
| *L. simplex* | 680 000 | 340 000 |
