# Supplementary material for: Coalescent Simulations Reveal Hybridization and Incomplete Lineage Sorting in Mediterranean Linaria
Source: PLoS One. 2012 Jun 29;7(6):e39089. doi: 10.1371/journal.pone.0039089 (PMC3387178; doi:10.1371/journal.pone.0039089)
Supplement: Table S3 — Assignment of genes to label 1 (L1) or label 2 (L2) in the multilabelled species tree analysis ( Fig. 5 ). (DOCX) [file pone.0039089.s004.docx]

**Table S3.** Assignment of genes to label 1 (L1) or label 2 (L2) in the multilabelled species tree analysis (Fig. 5).

| **Presumed hybrid** | **ITS** | **AGT1** | **cpDNA** |
| --- | --- | --- | --- |
| *L. glauca ssp. olcadium* | L1 | L2 | L1 |
| *L. orbensis* | L1 | L1 | L2 |
| *L. amethystea* ssp. *amethystea* | L1 | L2 | L1 |
| *L. cuartanensis* | L1 | L2 | L1 |
| *L. tursica* | L1 | L1 | L2 |
| *L. oblongifolia* ssp. *oblongifolia* | L1 | L2 | L1 |
| *L. alpina* | L1 | L1 | L2 |
| *L. filicaulis* | L1 | L1 | L2 |
| *L. saturejoides* ssp. *saturejoides* | L1 | L2 | L1 |
| *L. propinqua* | L1 | L2 | L2 |
