# Supplementary material for: Coalescent Simulations Reveal Hybridization and Incomplete Lineage Sorting in Mediterranean Linaria
Source: PLoS One. 2012 Jun 29;7(6):e39089. doi: 10.1371/journal.pone.0039089 (PMC3387178; doi:10.1371/journal.pone.0039089)
Supplement: Methods S1 — Supplemental methods. (DOCX) [file pone.0039089.s005.docx]

**Supporting Information**

**Methods**

**Nomenclatural changes required**

We included in the present paper two samples of the “*Linaria verticillata* group” [1]: *L. verticillata* ssp. *anticaria* (Boiss. & Reut.) L. Sáez & M.B Crespo – Spain: Málaga. El Torcal de Antequera, J.L. Blanco-Pastor (33JB09) – and *L. verticillata* ssp. *cuartanensis* (Degen & Hervier) L. Sáez & M.B. Crespo – Spain: Albacete. Yeste, P. F. Cannon et al. (2009.12.35 (RNG)). L. Sáez & M.B. Crespo considered that the “*Linaria verticillata* group” is formed by races that appeared as a result of geographical speciation from a southern Iberian common ancestor. As (i) taxonomic delimitation of this particular group has been controversial and (ii) non-monophyly was obtained for both samples used (see ITS and AGT trees in Fig. 1) such samples were treated here as independent taxa. Further, a possible hybrid origin or introgression was detected in the *L. verticillata* ssp. *cuartanensis* sample, thus not supporting such a simple geographical speciation model for this taxa as discussed by L. Sáez & M.B Crespo [1]. In consequence both samples of the “*Linaria verticillata* group” were herein circumscribed in a taxonomical rank at species level which we considered more appropriate: *L. anticaria* Boiss. & Reut. [2] was used instead *L. verticillata* ssp. *anticaria* whereas a nomenclatural change was required for *L. verticillata* ssp. *cuartanensis*:

***L. cuartanensis*** (Degen & Hervier) Fern. Casas ex. Blanco-Pastor, **comb. et stat. nov.**

**≡** *L. anticaria* ssp. *cuartanensis* Degen & Hervier in Bull. Acad. Int. Géogr. Bot. 15: 115 (1905) **[basionym]**

*L. anticaria* var. *cuartanensis* (Degen & Hervier) Degen in Bull. Acad. Int. Géogr. Bot. 16: 207 (1906)

*L. cuartanensis* (Degen & Hervier) Fern. Casas, in sched. 1976 (MA 348603, MA 348635), nom. inval.

For the same reason as discussed above, a similar taxonomical delimitation (rank at species level) was applied for the “*Linaria alpina* group” formed by the samples *L. alpina* (L.) Mill. ssp. *alpina* – Spain: Huesca. Formigal, S. Martín Bravo (571SMB05) – and *L. alpina* ssp. *filicaulis* (Boiss. ex Leresche & Levier) M. Laínz – Spain: León. Pico Tres Provincias, C.M. Romero Rodríguez (769084 (MA)). Those samples were then considered as *L. alpina* (L.) Mill. and *L. filicaulis* Boiss. ex Leresche & Levier [3].

Further molecular analyses not performed here including additional samples would be valuable to establish a clear taxonomic circumscription of the “*Linaria verticillata” and the “Linaria alpina”* groups.

**Coalescent Simulations**

The analysis proceeded as follows: (1) Infer gene trees including branch lengths in MrBayes 3.1.2 using allele data. (2) Draw a pool of trees from the stable posterior distribution and convert these to chronograms in r8s 1.71. (3) Scale the chronograms into generations in Mesquite 2.6 using previous date estimates and generation time of the species. (4) Derive mutation rates per generation (µ) in cpDNA using the average number of pairwise differences (D_xy_) between two species and their divergence time estimates. (5) Estimate allelic diversity (θ_w_) in one species using DNAsp 5.10. (6) Derive the effective population size (*N_e_*) via θ_w_ = 2µ*N_e_*. (7) With the scaled chronograms and *N_e_*, simulate under the coalescent new “gene trees” using the tool “coalescent contained within the current tree” in Mesquite 2.6. (8) Generate a distribution of tree to tree distances (symmetric distance in PAUP*) for each observed gene tree and corresponding simulated trees (baseline distribution), as well as among the gene trees (observed distribution), (9) Compare these distributions to determine whether significant differences exist among the observed gene trees in light of a lineage sorting null hypothesis.

Additional details for each step are now described.

For step 1, we used the haplotype trees of (AGT1 intron, ITS and cpDNA (rpl32-trnL^UAG^ and trnS-trnG intergenic spacers) inferred using MrBayes 3.1.2 [4].

For step 2, we drew 20 trees from the stable posterior distribution in order to account for uncertainty in our gene tree estimation. These trees were topologically representative of the complete Bayesian Analyses for each locus (consensus topologies of using most incongruent alleles are shown in Supplementary Figure 1), were representative of the entire post-burnin distribution of parameter values when compared using Tracer (e.g., produced overlapping 95% HPD estimates for log likelihood, posterior and other parameters). We converted the trees into chronograms using the penalized likelihood function [5] implemented in r8s [6] in order to have branch lengths estimate time in each gene tree. Cross-validation to find the optimal smoothing parameter (10^k^) was done using increments of k of 0.1, from k= -3 to 3 (using two random trees from the stable posterior distribution of each gene). Each chronogram was then trimmed to contain only a single allele from each individual, using Mesquite (Maddison and Maddison, 2006) to delete terminals while maintaining appropriate branch lengths. The alleles remaining were chosen to maximize topological incongruence among the loci, because several loci with alleles in the same clade do not introduce incongruence beyond lineage sorting and thus require no further explanation.

For step 3, chronograms obtained in r8s were based on a calibration point of the divergence between *Antirrhinum and Linaria* of 13.33 Ma (5% CI) (Vargas et al., unpublished). In Mesquite we scaled the branch lengths of the trees to convert them from units of time into units of generations. That was done by using the ultrametric trees, which implicitly assume equal generation times on all lineages, and setting the branches for annual species (and all branches in clades with only annuals) to have an “annual” scale (a generation time of one year). This assumes an early change to this state. The rest of the tree was treated as having the perennial state with a generation time of three years (generations divided by 3). Despite the impossibility in obtaining an ancestral state for annuality or perenniality (due to the incongruence among gene trees) with current methods, by doing this at least we could differentiate the generations scale (branch length scale) between the known annual species and the rest of the tree. By assuming perenniality in the rest of the tree we presented trees with shorter internal branches (fewer generations) for the coalescent simulations than if considering annuality, thus favoring the null hypothesis of lineage sorting alone to explain the gene tree incongruence (see below). In this way we are being conservative with resepect to the nul hypothesis.

The species having an “annual” scale were: *L. albifrons, L. flava, L. triphylla, L. laxiflora, L. warrionis, L. haelava, L. joppensis, L. arvensis, L. micrantha, L. simplex, L. propinqua, L. oblongifolia, L. saxatilis, L. bipunctata, L. tursica, L. glauca, L. saturejoides, L. bubanii, L. munbyana, L. filicaulis., L. badalii, L. amethystea, L. spartea, L. gharbensis, L. orbensis and L. chalepensis.*

For step 4, we calculated the mutation rate (μ_gen_), where μ_years_=D_xy/sites/years_/2 and μ_gen_ is obtained after scaling μ_years_. We calculated the average number of pairwise differences (D_xy_) between *L. elegans* (25 individuals from 25 different populations; trnS-trnG, rpl32-trnL and trnK-matK markers, Fernandez-Mazuecos, under review) and *L. cuartanensis* (1 individual, 1 population) using DNAsp [7]. Mutation rate per generation was obtained by dividing μ by the younger limit of the 95% HPD of divergence time (in years) between *L. elegans* and *L. cuartanensis* again, to favour the null hypothesis.

For step 5: Theta per sequence [θw, an estimate of the population mutation rate under the neutral model; 8] was also estimated using DNAsp [7] over the 25 *L. elegans* individuals.

To explore the influence of N_e_ estimates on the coalescent simulations, we also calculated N_e_ using other two *Linaria* species. As the range size of a species seems to be highly correlated with its N_e_ [see examples in 9], we used two more species: *L. glacialis* (100 individuals from 10 populations, rpl32-trnL and rps16-trnK^UUU^) and *L. simplex* (13 individuals from 13 populations, rpl32-trnL, trnS-trnG and trnL-trnF), with extreme lower and higher range sizes respectively. *Linaria elegans* occupies the northern part of the Iberian Peninsula, *L. glacialis* is a species that is restricted to a narrow altitudinal gradient on mountain and ridge tops in the Sierra Nevada range (Spain) and *L. simplex* is one of the few *Linaria* species with a widespread distribution, been present all over the Mediterranean basin.

For step 6, N_e_ was derived using θ_w_ = 2*N_e_*μ (2 for plastidial genes of diploid hermaphroditic species). We multiplied by 2 in order to apply the obtained values to nuclear sequence data. N_e_ values used in the coalescent simulations are shown in Supplementary Table 1.

For step 7, simulations on the set of chronograms were done in Mesquite assuming the above-mentioned *N_e_* values for all species and their common ancestors, with 100 simulated trees produced for each input tree. Input trees were the 20 trees from the stable posterior distribution of each MrBayes analysis converted to chronograms in r8s and pruned to contain one allele (the most differentiated allele in respect to the other loci).

For step 8, nexus files were made that included the trees to be compared to one another and an analysis block describing an analysis of symmetric distance [10]. Each of the 20 trees drawn from the stable posterior distribution from the BA was placed at the beginning of the block of 100 simulated trees that arose by coalescent simulation. The first tree for each block was then compared to the remainder (using PAUP* commands “treedist metric=symdiff fromtree=1”). All 2000 distances generated this way (per locus) were pooled for subsequent steps. Distances obtained represented the “baseline distribution of tree distances”. The 20 BA trees for each locus were pairwise compared to one another in a similar manner and also pooled for each pairwise comparison. Those distances represented the “observed distribution of tree distances”. The distributions of distances were plotted in Excel (Microsoft). The null distribution is the distribution of values of the difference between the lower 95% HPD of the observed distribution and a critical value of the base line distributions (which depends on the number of loci used), under lineage sorting alone, and which was derived previously by simulation [11]. The critical value is chosen such that 5% of values of the null distribution fall above zero. However, the null distribution itself is not generated here.

For step 9, by doing pairwise comparisons, the null hypothesis of lineage sorting alone was rejected if none of the tree-to-tree distances (observed distributions), for a certain gene tree within their 95% HPD, overlapped the base line distribution of the gene used.

**References**

1. Sáez L, Crespo MB (2005) A taxonomic revision of the Linaria verticillata group (Antirrhineae, Scrophulariaceae). Botanical Journal of the Linnean Society 148: 229-244.

2. Boissier E, Reuter GF (1852) Pugillus plantarum novarum Africæ borealis Hispaniæque australis: Ex Typographia F. Ramboz et Socii.

3. Leresche LF, Levier E (1879) Decas Plantarum Novarum in Hispania Collectarium. Journal of botany, British and foreign 17: 200.

4. Ronquist F, Huelsenbeck JP (2003) MRBAYES 3: Bayesian phylogenetic inference under mixed models. Bioinformatics 19: 1572-1574.

5. Sanderson MJ (2002) Estimating Absolute Rates of Molecular Evolution and Divergence Times: A Penalized Likelihood Approach. Mol Biol Evol 19: 101-109.

6. Sanderson MJ (2003) r8s: Inferring absolute rates of molecular evolution and divergence times in the absence of a molecular clock. Bioinformatics 19: 301-302.

7. Rozas J, Sánchez-DelBarrio JC, Messeguer X, Rozas R (2003) DnaSP, DNA polymorphism analyses by the coalescent and other methods. Bioinformatics 19: 2496-2497.

8. Watterson GA (1975) On the number of segregating sites in genetical models without recombination. Theoretical Population Biology 7: 256-276.

9. Gossmann TI, Song B-H, Windsor AJ, Mitchell-Olds T, Dixon CJ, et al. (2010) Genome Wide Analyses Reveal Little Evidence for Adaptive Evolution in Many Plant Species. Molecular Biology and Evolution 27: 1822-1832.

10. Penny D, Hendy MD (1985) The Use of Tree Comparison Metrics. Systematic Zoology 34: 75-82.

11. Maureira-Butler IJ, Pfeil BE, Muangprom A, Osborn TC, Doyle JJ (2008) The reticulate history of *Medicago* (Fabaceae). Systematic Biology 57: 466-482.
